# Ischemic Heart Disease in Nigeria: Exploring the Challenges, Current Status, and Impact of Lifestyle Interventions on Its Primary Healthcare System

**DOI:** 10.3390/ijerph19010211

**Published:** 2021-12-25

**Authors:** Daniel A. Nnate, Chinedum O. Eleazu, Ukachukwu O. Abaraogu

**Affiliations:** 1 Department of Nursing and Community Health, School of Health and Life Sciences, Glasgow Caledonian University, Glasgow G4 0BA, UK; 2 Department of Public Health, Faculty of Health and Social Care, University of Chester, Chester CH1 1SL, UK; 3 Department of Chemistry, Biochemistry & Molecular Biology, Alex Ekwueme Federal University Ndufu-Alike, Abakaliki 482131, Ebonyi State, Nigeria; chinedum.eleazu@funai.edu.ng; 4 Department of Medical Rehabilitation, University of Nigeria, Enugu 410001, Enugu State, Nigeria; ukachukwu.abaraogu@unn.edu.ng; 5 Department of Physiotherapy and Paramedicine, School of Health and Life Sciences, Glasgow Caledonian University, Glasgow G4 0BA, UK

**Keywords:** behavioural change, cardiovascular diseases, diet, health promotion, Nigeria, lifestyle, physical activity, Universal Health Coverage, sub-Saharan Africa

## Abstract

The burden of ischemic heart disease in Nigeria calls for an evidence-based, innovative, and interdisciplinary approach towards decreasing health inequalities resulting from individual lifestyle and poor socioeconomic status in order to uphold the holistic health of individuals to achieve global sustainability and health equity. The poor diagnosis and management of ischemic heart disease in Nigeria contributes to the inadequate knowledge of its prognosis among individuals, which often results in a decreased ability to seek help and self-care. Hence, current policies aimed at altering lifestyle behaviour to minimize exposure to cardiovascular risk factors may be less suitable for Nigeria’s diverse culture. Mitigating the burden of ischemic heart disease through the equitable access to health services and respect for the autonomy and beliefs of individuals in view of achieving Universal Health Coverage (UHC) requires comprehensive measures to accommodate, as much as possible, every individual, notwithstanding their values and socioeconomic status.

## 1. Introduction

Attaining global sustainability and health equity has been the focus of modern healthcare systems as it aims to embrace a universal approach to promoting the optimum health of humans, animals as well as the environment [1]. This calls for an interdisciplinary approach to uphold the holistic health of individuals, which is inclined towards improving their psychological and physical wellbeing through the equitable access to healthcare, healthy diet, green industrialisation, and respect for individual values [2]. The recognition of extreme poverty, malnutrition, access to clean water, maternal and child mortality, infectious diseases (such as HIV, hepatitis, tuberculosis, and malaria) as the major burden of low- and middle-income countries (LMICs) led to the introduction of the millennium development goals (MDGs) to eradicate these challenges by the year 2015 [3].

According to Buse and Hawkes [4], the implementation of MDGs paved the way for increased aid flow to these deprived areas, but there was no evidence of its impact on health policies to promote positive health outcomes. While these goals were yet to be achieved by 2015, there was also an upsoar of zoonotic and non-communicable diseases (NDCs), unemployment, gender inequality, climate change from increased deforestation and use of fossil fuels, and inadequate healthcare delivery due to insufficient health workforce all over the globe [5,6]. These events saw to the introduction of sustainable development goals (SDGs) to incorporate economic growth and environmental protection that could be sustained over a longer period.

The introduction of SDGs saw a paradigm shift from prioritising the health of poor nations to the entire world, taking into consideration the impact of NCDs, industrialisation, migration, antimicrobial resistance, and the re-emergence of infectious diseases on humans [6]. While globalisation brought along economic and health benefits that are now experienced all over the world, the prevalence of NCDs, such as ischemic heart disease (IHD), has risen in LMICs, where more than three-quarters of the world’s population now inhabit [7,8]. The current trend in epidemiologic transition and its effect on the total disease burden of cardiovascular diseases in Nigeria are explored in this paper, with an emphasis on public health interventions aimed at promoting a positive lifestyle.

## 2. Nigerian Healthcare, Demography, and Epidemiologic Inclination towards IHD

The less developed countries of Africa suffer multiple burdens of disease as a result of rapid urbanization and the adoption of a Western lifestyle [7,9]. Ischaemic heart disease, however, is caused by the build-up of fatty deposits over a long period in the blood vessels by the process known as atherosclerosis [10]. Atherosclerosis is a systemic disease, affecting not only the coronary arteries, but also the aorta, the cerebral, renal, mesenteric and limb arteries, with symptoms and signs dependent on the location and severity of vascular stenosis [10]. Therefore, patients with atherosclerosis are at risk of developing acute coronary events, such as myocardial infarction, cardiac arrhythmias (atrial fibrillation being the most common sustained cardiac arrhythmia in adults), and eventually, heart failure. They may develop stroke or peripheral arterial disease or chronic kidney disease as well.

Ischaemic heart disease, the consequence of coronary atherosclerosis, has modifiable and non-modifiable risk factors [11]. Among modifiable risk factors, of utmost importance are high blood pressure, hypercholesterolemia, diabetes mellitus, and obesity. Symptoms, such as chest pain, breathlessness, and heart palpitations, are clinical manifestations of the progressive narrowing of the coronary arteries by atherosclerotic plaques. Ischaemic heart disease is responsible for about 7.4 million deaths globally, contributing to a third of all deaths on Earth, and is the most common among all NCD-related deaths [12]. This figure is further projected to increase to about 23.6 million people by 2030 [13]. Although IHD is believed to be associated with the ageing population, young adults also make up a greater percentage of these deaths in developed countries and developing countries, such as the sub-Saharan African nations [7,8,13].

According to reports by the World Bank [14], Nigeria is the most populous country in Africa with 190 million people, making up about 50 per cent of the population of West Africa. Within the country, the effects of urbanization and the migration of individuals to cities have further contributed to the disparities in lifestyle and health between the rural and urban areas [7]. Similar to other nations of the world, the Nigerian health system also places much priority in terms of financing and healthcare delivery on curative and preventive measures [15]. Yet, the nation’s healthcare is regarded as one of the poorest compared to other West African countries, with a lower gross domestic product (GDP) due to the low life expectancy of its citizens [16].

**Figure 1:**
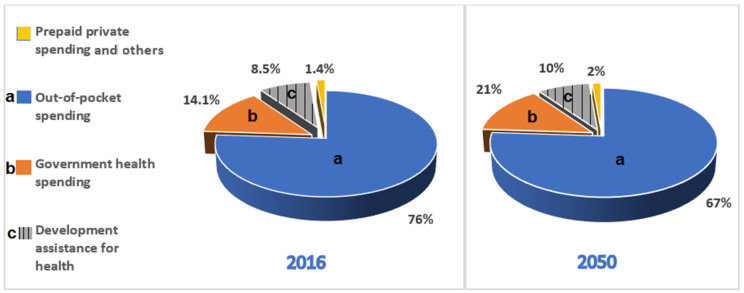
Expected healthcare expenditure in Nigeria, based on past growth [21].

In view of achieving the Universal Health Coverage (UHC), there has been a huge reliance on public funds in maintaining health services [17]. This implies that a substantial amount of spending on health per capita by each nation may be required to meet this goal. Therefore, in developed countries, it may be considered that the government prioritization of health contributes to the increased life expectancy and economic growth that is realized. Varghese et al. [18], in their analysis, also proposed putting in place interventions within primary care for the management of NCDs across health systems globally. A probable explanation for this could be due to the increasing surge in the number of people utilizing various inpatient and outpatient services because of NCDs, such as cardiovascular disease, diabetes, chronic kidney disease, and respiratory disorders. It was further pointed out that a primary healthcare system that is more focused on the prevention and management of NCDs could reduce over half the total cause of mortality worldwide [18,19]. Therefore, with health systems driven towards reducing the burden of NCDs, it could be possible to end millions of premature deaths from IHD in sub-Saharan African nations and other LMICs. However, while interventions to mitigate the prevalence of NCDs at the primary care level requires a significant financial commitment by the government, Chang et al. [20] suggest that the less spending in health per capita in low-income and sub-Saharan African countries could be a result of the government’s priority being more inclined to economic development with less attention paid to health, which may be a flawed strategy. Though the government contributes to about 27 per cent of Nigeria’s healthcare expenditure, the deficit is then covered by the private health sector and the supplementary intervention of non-governmental organizations [16]. The poor investment in the health sector creates a huge burden in the form of “out-of-pocket” expenses for Nigerian citizens, as highlighted in Figure 1. Out-of-pocket payment for health services is considered a major barrier to seeking immediate treatment, which further contributes to poor health outcomes among individuals.

**Figure 2:**
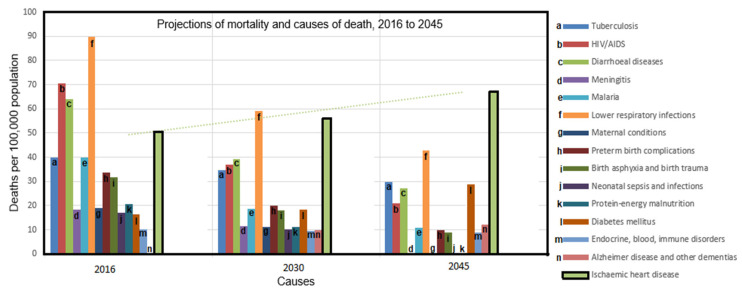
The projection of mortality and cause of death between 2016 to 2045 shows an exacerbation of IHD in Africa [13].

Before proceeding to analyze the prevalence and risk of cardiovascular diseases in the country, it is important to note that Nigeria has its share of the global burden of disease and is also far from achieving most of the SDGs, similar to other LMICs in Africa (Figure 2). Currently, about 1.9 million people are living with HIV with an adult prevalence rate of 1.5 per cent [14,21]. Nigeria also has a high infant and maternal mortality rate of 63.3/1000 live births and 917/100,000 live births, respectively, from preeclampsia, malaria, and vaccine-preventable diseases [22]. With a low life expectancy of 54 years, coupled with a growth rate of about 2.5 per cent and ages 0–14 occupying 43.8 per cent of the population, it could be considered that life expectancy is reduced at childhood [14]. Furthermore, while individuals between 15–65 years make up 52.4 per cent of the population, there is an increased vulnerability to NCDs, such as diabetes and cardiovascular diseases, among this age group [13,23].

There is a complex interaction with the HIV infection and the increased prevalence of IHD has been well recognised in the affected population [24,25]. There is some evidence that antiretroviral therapy predisposes people living with HIV to further cardiovascular risks, such as dyslipidemia, insulin resistance, and endothelial dysfunction [24,25]. The role of HIV-related inflammation and immune activation in the increased risk of IHD cannot be overstated. Recent research has revealed that the prognosis of IHD in people living with HIV is also influenced by other variables, particularly socioeconomic and environmental factors, most of which are poverty related [24]. Considering that about 70% of the world’s HIV-positive population lives in sub-Saharan Africa, a great number of people on antiretroviral therapy, routine cardiovascular risk assessment, regardless of therapy status, is required to prevent future cardiovascular events.

The increased prevalence of IHD may be due to the unhealthy lifestyles adopted from adolescence to adulthood that tend to put individuals at a higher risk. Lifestyle and underlying conditions that make individuals more suspectable to IHD include tobacco use, unhealthy diet, physical inactivity, obesity, and excess alcohol intake [26,27]. It is believed that most people diagnosed with type 2 diabetes mellitus will develop a cardiovascular disorder at a later stage in the disease prognosis. Udenze and Amadi [28] have identified type 2 diabetes mellitus and obesity as major risk factors in the development of IHD in Nigeria. With a prevalence of 4.3 per cent, it was further estimated that over 5 million people would be diagnosed with type 2 diabetes mellitus by 2030 [28]. The incidence of diabetes is linked to insulin resistance that comes with ageing in susceptible adults, but the consumption of high-calorie diets also puts a strain on the glycogenesis/glycogenolysis pathway as glucose metabolism may also favour de novo lipogenesis leading to excess weight due to its storage as body fat.

Hyperlipidaemia due to the increased consumption of a high-calorie diet is indicated as a metabolic risk factor of IHD. There is also evidence of insulin resistance from the conversion of non-esterified fatty acids of the adipose tissue [29]. Mayo [30] further highlighted the cardiovascular risk posed by individuals from Black-African ethnic origin who are unable to store fats in less metabolically active adipose tissues of the thighs and buttocks, thereby resulting in a central distribution of free fatty acids, which further increases the lipid content in circulation. Hence, the need to strengthen the evidence is required in order to adopt a logical approach to addressing this key issue. The inclination of the country’s GDP towards crude oil further exacerbates the prevalence of diabetes and IHD due to less prioritisation of agricultural products and the low intake of freshly grown fruits and vegetables [31]. While this may have led to a reduction in the daily recommended intake of fruits and vegetables with a concomitant increase in the incidence of cardiovascular diseases, the changes brought about by the food retail industry tend to promote an unhealthy diet among inhabitants of LMICs.

According to the World Health Organization [32], three-quarters of the 1.1 billion worldwide tobacco users live in LMICs. Although the prevalence of tobacco-related deaths appears to be increasing in sub-Saharan African countries, Nigeria, being one of the leading tobacco markets in Africa, is more predisposed to tobacco-related IHD mortality with an increasing prevalence of four percent every year [26,33]. Hence, while there is a reported decline in tobacco use worldwide, the high prevalence in sub-Saharan African countries, such as Nigeria, may be related to the government’s weak policy on tobacco firms, owing to its contribution to the country’s international trade and commerce.

Moreover, the harmful use of alcohol is also considered a significant public health burden in Nigeria. Globally, alcohol consumption per capita was projected to be 6.3 litres of pure alcohol per person aged 15 years and above in 2015; however, in Nigeria, it was estimated at 11.3 litres, which is almost double the global estimate [34]. While the frequency of alcohol use tends to rise with individuals of higher socioeconomic status, a probable increase amongst people of lower socioeconomic status may be related to the poor level of education and its usage as a coping mechanism from stress. The effect of little or no alcohol consumption on the risk of developing IHD has been deliberated by researchers over a long period [35,36]. This further creates a medical and social consequence for both healthcare professionals as well as consumers regarding alcohol intake, thus leading to a cycle of what should be regarded as little or too much.

## 3. Challenges to the Integration of IHD Prevention Strategy into the Healthcare System of Nigeria

The prevalence of IHD is not only a problem associated with Western countries, but it is also increasingly becoming a problem that greatly impacts the economy of most sub-Saharan African countries [8,34]. The multiple burdens of disease in Nigeria are evident in the uniqueness and pattern of comorbid occurrence of communicable and non-communicable diseases, in addition to the increased prevalence of social inequalities between the rural and urban areas [37]. While the GDP and life expectancy at birth determines the affluence and health of individuals in a nation, the Nigerian health system fails to compensate for the individuals unproportionately affected by social inequalities [38]. Although there is a high prevalence of IHD in affluent areas due to unhealthy diets and sedentary lifestyles, research has also shown a direct relationship between IHD, adverse childhood experience, poverty, and low socioeconomic status [39,40]. Thus, promoting equity and fairness within society becomes another key necessity for reducing health inequalities and providing equal opportunities for individuals to flourish. Ischemic heart disease may be considered a model health disorder that is associated with the social determinants of health as the likelihood of being affected by the condition depends on a variety of factors, such as stress, economic and social environment, childhood experience, nutritional status, lifestyle, and individual behaviour.

Nigeria has a huge deficit in healthcare facilities and personnel, which is further aggravated by the low percentage of GDP invested in healthcare [16,20]. While infectious diseases seemed to be the focus of Nigeria’s health agenda as observed from most public health research and funding, non-communicable diseases and their associated risk factors have been neglected in previous public health intervention programs [41]. Gupta and Yusuf [42] further stressed that the lack of expertise on cardiovascular diseases in sub-Saharan Africa intensifies the problem as most health professionals specialized in infectious and tropical diseases, which were the focus of most advanced public health courses in accordance with the MDGs. This becomes evident in its poor diagnosis and management, which creates little awareness for the government regarding the need to make changes to the health system. Owing to the inadequate sensitization at the community level and poor knowledge of disease prognosis, patients may lack the ability to self-care and may often present late with symptoms at hospitals [43]. Hence, this necessitates the need to raise public awareness and interventions aimed at minimizing further strains on the health system, which will be explored further.

The enquiry into health inequalities between people of different socioeconomic statuses and how it influenced their health led to a breakthrough in reducing morbidity, improved mental health and general wellbeing in European countries [44,45]. It became evident that health inequalities result mainly from social inequalities that take the form of inaccessibility to a healthy standard of living, poor working conditions, inadequate nutrition, and poor health promotion strategies to encourage early childhood development and disease prevention [46]. However, in LMICs, such as the sub-Saharan African nations, many investigations into the causes of health inequalities are deficient due to the ill-structured health system [42,47]. Thus, it becomes almost impossible to devise strategies to mitigate the health inequalities caused by these social determinants of health.

## 4. Health Interventions to Address the Prevalence of IHD in Nigeria

Behavioural change may be considered as a public health intervention for disease prevention aimed at altering people’s habits and attitudes. While the focus of health promotion is empowering individuals to gain control over the factors that affect their health, it is believed that human behaviour, much of which is influenced by cultural, social, economic factors, may either stall or promote disease prevention [40,45]. The concept of behavioural change has been studied as far back as the late 20th century, with the development of theories to promote its use in healthcare [48]. This interactive process relies on evidence-based models to systematically promote positive behaviours within various social settings by assessing the underlying factors that encourage the receptivity of the target group to ideas, such as their health literacy level and ability to identify the credibility of health information and adherence to the intervention.

The behavioural risk factors of IHD include a poor diet, inadequate physical activities, tobacco use, excess alcohol intake, and a sedentary lifestyle [49,50]. In addition, the presence of underlying conditions, such as obesity, hypertension and dyslipidaemia, were also associated with individuals of a high social class who live in urban areas [51,52]. While it is believed that a sedentary lifestyle is prominent in high-income countries (HICs) compared to less affluent nations, there are limited studies to accept such claims with regards to developing nations, such as sub-Saharan Africa countries [53,54]. However, it has been reported that physical inactivity among individuals in LMICs is no different from those in HICs [54,55]. The decline in physical activity could be because of individuals carrying out their daily activities from the comfort of their homes.

People make informed decisions based on the evidence available to them, and their intention to change happens to be within their control [56]. Therefore, following a systematic approach to alter a person’s behaviour to improve their health outcome may serve as a basis for self-care through the prompt recognition of symptoms and timely help-seeking [57]. However, owing to the presence of multiple factors that are likely to impact a person’s behaviour, a complete change in health-related behaviour may not be feasible due to situations in which the individual has little or no control over the factors affecting the actual performance of behaviour [56]. Therefore, interventions that could mitigate these factors may come into play, such as laws and policies that limit people’s exposure to certain risks [45]. For instance, placing a levy on commodities, such as sugar, tobacco, and alcohol, in developing countries may deter consumers from indulging in such risky behaviour, which in turn limits their chances of developing NCDs.

Recently, there has been an increased awareness of IHD in Nigeria. Several educational models of IHD prevention, including population-wide and individual approaches targeting either individual risk factors or multiple risk factors, could be incorporated into the teaching curriculum at all levels, especially primary and secondary levels. Epidemiological evidence has been often used by government and health policymakers when developing guidelines and health education interventions [58]. Furthermore, social and behavioural change communication with mass media and health education interventions were found to be cost effective for reducing the risk factors of IHD at the population level [58]. An initiative by the Federal Government of Nigeria to regulate the production, sale, and promotion of tobacco products led to the implementation of [59] to minimise the effect of tobacco on the prevalence of IHD. The Federal Ministry of Health (FMoH) has also developed interventions aimed at strengthening the management of NCDs in primary healthcare by promoting physical activities and a healthy diet among individuals. This is further accompanied by dietary modifications, physical activity, and behavioural change measures on tobacco and alcohol cessation [38,60]. According to a recent report by the World Health Organization [61], there has also been a move to collaborate with the FMoH to implement essential NCD interventions across primary health care along with various national multi-sectoral action plans on NCDs. This partnership is aimed at eradicating poverty and social inequalities emanating from IHD and other NCDs, with much effort being made to increase government spending in line with the Universal Health Coverage.

While most interventions predominantly target modifiable risk factors for IHD, such as diabetes, hypertension, obesity, and hypercholesterolemia, Ebireri et al. [60], in their study, concluded that it was almost unlikely to determine the effectiveness of these interventions across Nigeria’s diverse population due to variations in lifestyle and risk factors. Most interventions for IHD often integrate complementary and alternative medicine (CAM) into behavioural change for the management of NCDs, such as reduced sugar and salt intake, use of modified cooking oil (soya bean oil and mmega-3 fatty acids), and dietary supplements [62,63]. While there has been an increasing amount of literature on CAM approach to managing IHD, Park and Canaway [64] further suggested that integrating CAM into the healthcare system is likely to promote equitable access to health services and respect for the autonomy of service users, and further maximize the possibilities of achieving Universal Health Coverage.

The uniqueness of the Nigerian healthcare system becomes apparent as it incorporates the therapeutic impact of herbal medicine and faith healing with current medical practices as not all individuals place full confidence in conventional medicine [65,66]. Anand et al. [67] further emphasized that alternative interventions that consider ethnicity and culture would further reduce the dependence on modern health services, which may not be easily accessible to certain individuals. Owing to the multicultural nature, beliefs, and varying perceptions of Nigerians towards managing IHD, research into innovative strategies to support primary health care becomes essential. This also creates an avenue to promote indigenous medical practices that exist within the various cultural groups.

## 5. Conclusions

The dawn of epidemiologic transition due to changes in lifestyle is well established all over the world, and the prevalence of IHD in Nigeria, compared to other developed nations, seems to follow a similar trend as a result of globalization. This article offers an insight into the challenges of mitigating the risk of cardiovascular diseases in Nigeria. While the advancement in technology may have played a major role in the poor lifestyle choices among individuals, it is certain that an unhealthy diet, physical inactivity, and poor socioeconomic status predisposes individuals to cardiovascular diseases. In view of all that has been mentioned so far, one may suppose that further research on the impact of beliefs and ethnicity on Nigeria’s primary health care system is especially important when individual behaviour, the existence of complementary therapies, and increased out-of-pocket expenses act synergically to influence health service delivery.

After 5 years since the start of the realization of the SDGs agenda, the evidence presented in this paper suggests that tackling the burden of IHD in Nigeria warrants an innovative and holistic approach. In this regard, the possibility of creating sustainable healthcare interventions for managing the rising pandemic of chronic cardiovascular disorders and other NCDs across the nation will only be feasible if there are strategies and policy measures put in place to promote behavioural change across indigenous cultures and, furthermore, meet the health literacy needs of less affluent societies. Hence, comprehensive measures to mitigate cardiovascular risk factors should accommodate as much as possible every individual, notwithstanding their beliefs and socioeconomic status.

## Data Availability

Not applicable.
